# Genetic ancestry of families of putative Inka descent

**DOI:** 10.1007/s00438-018-1427-4

**Published:** 2018-03-03

**Authors:** José R. Sandoval, Daniela R. Lacerda, Marilza S. Jota, Ronald Elward, Oscar Acosta, Donaldo Pinedo, Pierina Danos, Cinthia Cuellar, Susana Revollo, Fabricio R. Santos, Ricardo Fujita

**Affiliations:** 1 Centro de Genética y Biología Molecular (CGBM), Instituto de Investigación, Facultad de Medicina Humana, Universidad de San Martín de Porres (USMP), Lima, Peru; 2 Laboratório de Biodiversidade e Evolução Molecular (LBEM), Instituto de Ciências Biológicas, Universidade Federal de Minas Gerais, Belo Horizonte, Brazil; 3 Limaq Publishing SAC, Lima, Peru; 4 Universidad Mayor de San Andrés (UMSA), La Paz, Bolivia

**Keywords:** Inkas, Panakas, Y-SNPs, Y-STRs, Y chromosome, MtDNA

## Abstract

This study focuses on the descendants of the royal Inka family. The Inkas ruled *Tawantinsuyu*, the largest pre-Columbian empire in South America, which extended from southern Colombia to central Chile. The origin of the royal Inkas is currently unknown. While the mummies of the Inka rulers could have been informative, most were destroyed by Spaniards and the few remaining disappeared without a trace. Moreover, no genetic studies have been conducted on present-day descendants of the Inka rulers. In the present study, we analysed uniparental DNA markers in 18 individuals predominantly from the districts of San Sebastian and San Jerónimo in Cusco (Peru), who belong to 12 families of putative patrilineal descent of Inka rulers, according to documented registries. We used single-nucleotide polymorphisms and short tandem repeat (STR) markers of the Y chromosome (Y-STRs), as well as mitochondrial DNA D-loop sequences, to investigate the paternal and maternal descent of the 18 alleged Inka descendants. Two Q-M3* Y-STR clusters descending from different male founders were identified. The first cluster, named AWKI-1, was associated with five families (eight individuals). By contrast, the second cluster, named AWKI-2, was represented by a single individual; AWKI-2 was part of the Q-Z19483 sub-lineage that was likely associated with a recent male expansion in the Andes, which probably occurred during the Late Intermediate Period (1000–1450 AD), overlapping the Inka period. Concerning the maternal descent, different mtDNA lineages associated with each family were identified, suggesting a high maternal gene flow among Andean populations, probably due to changes in the last 1000 years.

**Electronic supplementary material:**

The online version of this article (10.1007/s00438-018-1427-4) contains supplementary material, which is available to authorised users.

## Introduction

*Tawantinsuyu* was the climax of approximately six millennia of autochthonous cultures in the Central Andes since the *Norte Chico* culture. The Inkas ruled *Tawantinsuyu* for approximately 150 years (Marsh et al. 2017) until the arrival of the Europeans in 1531. The first tribal settlement of the Inkas in the Cusco valley in Peru probably dated to the twelfth century, and their empire expanded to a territory of approximately 1,800,000 km^2^ with the largest political system of pre-Columbian Americas. According to a genetic study (Sandoval et al. 2013a), the current population of the Central Andes represents the highest proportion of Native American ancestry, and most Andeans are probably direct descendants of the *Tawantinsuyu* people. Furthermore, other studies have ratified the cultural and genetic homogeneity among Andean populations in contrast to the highly heterogeneous eastern lowland Amazonian populations (Tarazona-Santos et al. 2001; Sandoval et al. 2016). These and other genetic studies (Llamas et al. 2016; Jota et al. 2016) have shown significant advances in recovering the Native American history; however, no study has focused on the descent of the royal Inka family.

Due to the haploid mode of inheritance, the Y-chromosome and mitochondrial (mt) DNA markers are widely used in reconstructing the genealogical history of populations. Genetic analysis of autochthonous Y-chromosome markers shows most South American populations with haplogroup Q (characterised by different sub-lineages) and other rare haplogroups, such as C-M217 (Jota et al. 2016). Simultaneous analysis of Y-chromosome single-nucleotide polymorphisms (Y-SNPs), which define a haplogroup or sub-lineage, and short tandem repeats (Y-STRs), which are used for paternal testing, familial genealogy and kinship identification, have allowed for the discrimination between sub-lineages among South American natives (Jota et al. 2011, 2016). Using multiple genotyping approaches, we identified informative SNPs exhibiting restricted Q haplogroup sub-lineages in South American populations (Jota et al. 2016). For example, the Q-Z19483 sub-lineage is associated with population movements of the Late Intermediate Period, as it is distributed in Central Andes, including the Altiplano region, and probably connected with the Inka expansion.

With respect to matrilineal inheritance, South American natives exhibit mainly four mtDNA haplogroups (A2, B2, C1 and D1), which comprise several sub-lineages and minor haplogroups, such as D4h3a (Brandini et al. 2017). The coalescence times for each Native American mtDNA haplogroup are estimated between 14 and 20 kya (Llamas et al. 2016; Brandini et al. 2017; Barbieri et al. 2017), suggesting that the first human settlers arrived in Americas by the end of the Pleistocene (Moreno-Mayar et al. 2018). Furthermore, comparisons of mtDNA haplogroup frequencies between ancient and modern South American populations show distinct regional patterns (Fehren-Schmitz et al. 2015; Barbieri et al. 2017). However, a general continuity in northern, central and southern parts of South America is apparent, with the North and Central Andes displaying high intra-population and low inter-population diversities with a high prevalence of haplogroup B2 due to greater effective population size and continuous gene flow (Valverde et al. 2016; Brandini et al. 2017). Coincidentally, the high prevalence area of the B2 lineage corresponds to the *Tawantinsuyu* region.

Information on lineages of the Inka nobility and rulers of *Tawantinsuyu* and their ancestral origins is limited to data gathered by the Spanish chroniclers, anthropologists and modern historians and becomes more uncertain and surrounded by myths as we go further back in time (Gamboa 1572; de la Vega 1609; Cobo 1653; Bauer 1991; Espinoza-Soriano 1997). Although the origins of the Inka family before their settlement in Cusco are still unknown, there are two major legends: (1) an older myth that the Inka ancestors originated from a cave in the district of Pacarictampu in the Province of Paruro, 50 km south of Cusco and (2) an imperial tale that the ancestors originated alongside the Sun at the *Isla del Sol* on the Bolivian side of Lake Titicaca at 380 km southeast of Cusco (Urton 2004). For many scholars, these alternative origin places are not contradictory and represent successive settlements of the original migration of the Inka ancestors (Espinoza-Soriano 1997; Bauer and Covey 2002; Cerrón-Palomino 2013).

While historical data have been published on families of putative Inka descendants (Amado-Gonzales 2009; Dunbar-Temple 2009), this information is available only for the colonial period until 1824. To identify the present-day patrilineal descendants of the Inka rulers, it is necessary to identify families that were recognised as Inka descendants before 1824 and to reconstruct family genealogies till date. At the start of the colonial period, the putative descendants of Inka rulers were recognised as nobles with rights, were not required to pay tributes and were exempt from forced labour. After the rebellion of Tupac Amaru II when the colonial government implemented political changes related to the native nobility, it became important to again prove noble ancestry. Several documents are available during the period 1780–1824 that not only identify noble families, but also contain data on ancestors and lineages.

The complex society of the pre-Columbian Central Andes is based on ‘*ayllu*’, a kinship system of families and clans that shared the same land and labour division for several generations (Espinoza-Soriano 1997). During the Inka Empire, new *ayllus* were founded by different rulers for political and administrative management of *Tawantinsuyu*, as well as for worship in the afterlife for coming generations. Pachacutec, the first Inka Emperor, was responsible for reorganising the state and imperial family. Ten imperial *ayllus*, or groups of rulers’ descendants, were distributed into two groups: five for lower (*hurin*) Cusco from former Manco Capac till Capac Yupanqui and five for higher (*hanan*) Cusco from Inka Roca till Tupac Yupanqui, the son and successor of Pachacutec (Rostworowski 2001; Zuidema 2007). During the Spanish rule around 1570, Viceroy Francisco de Toledo again reorganised the imperial descendants in Houses, and the Pachacutec *ayllu* was split into two (Supplemental Table 1) (Gamboa 1572; Rostworowski 2001; Amado-Gonzales 2003). By 1572, the descendants of the Inka royal family had properties assigned by the Spanish crown in six so-called parishes of ‘Indians’ around Cusco: Belén, Hospital de Naturales, San Blas, San Cristobal, San Sebastian and San Jerónimo. The parishes of Santiago and Santa Ana were reserved for other groups (Gamboa 1572). During colonial times, uninterrupted written records of the noble Inka descendants (known as Panakas) could be found on census, municipal, parochial and legal registers until 1824 (Amado-Gonzales 2003). With the formation of the Peruvian Republic, privileges were abolished and these descendants were no longer a separate group. Other Inka descendants were also registered in the Lake Titicaca Basin, including Azángaro, Capachica and Copacabana, on the Peruvian and Bolivian sides of Lake Titicaca (de León 1553; Espinoza-Soriano 1972). A few families have been traced during the colonial period (Amado-Gonzales 2003, 2005, 2009; Dunbar-Temple 2009); these may be used as subjects of research in the absence of mummies of the Inka rulers. Most mummies were probably destroyed by Corregidor Polo de Ondegardo in the late sixteenth century (Deza and Barrera 2001), whereas five that were displayed in the Hospital de San Andrés, Lima, disappeared in the first half of the seventeenth century. Moreover, no evidence has been recovered from the Convent of Santo Domingo in Cusco where the remains of the Inkas of Vilcabamba were buried. With the complete absence of genetic material of former rulers, present-day Panakas families are the only DNA source that could help gain insight into the origin of the Inka rulers.

In this study, we focused on the Panakas families to investigate whether they are related to each other by a patrilineal descent, and if they are genetically linked to populations from Lake Titicaca or to those from Pacarictampu. To tackle these questions, we compared Y-chromosome and mtDNA data from the Panakas with individuals from different provinces of Cusco, including the Pacarictampu district, and many Quechua- and Aymara-speaking populations from Peru (including populations from Lake Titicaca), Bolivia and Ecuador.

## Materials and methods

### Ethics statement and sampling

To reconstruct the genealogical trees of the identified families, all parish records for Cusco, records of eight parishes from the eighteenth and nineteenth centuries (available in the Archivo Arzobispal del Cusco and the parish churches), records from the Registro Civil del Cusco during 1900–1950 and all testaments and property transactions (available in the Archivo Regional del Cusco) have been revised. By 1800, 65 families were identified as being of noble Inka origin, of which 27 are still living in Cusco (Supplemental Table 2a and 2b). This part of the research has been done by Ronald Elward, and forms the basis for his master’s degree’ thesis in history at the UNMSM under the title “Los Incas Republicanos, la élite indígena cusqueña entre asimilación y resistencia cultural durante el Siglo XIX”, currently under way.

**Fig. 1:**
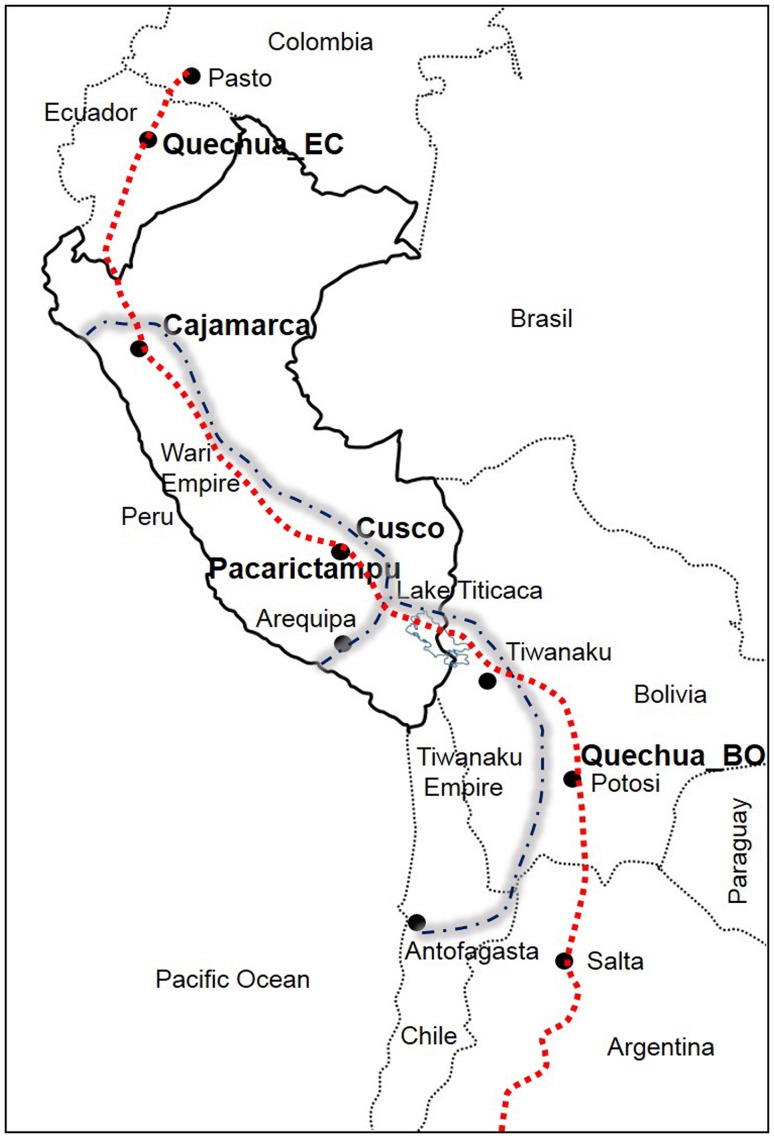
Map showing the reference locations of samples investigated in this study. Dashed lines that cross from Pasto to Peru, Bolivia and Argentina indicate a part of the ‘backbone’ of *Qhapaq Ñan* (Great Inka Road). Dashed and dotted lines show the geographical extension of the Wari and Tiwanaku Empires

A total of 19 individuals (18 men and 1 woman) from 12 different families with a documented lineage were tested. Tissue samples using buccal swabs were collected according to standard procedures. Written informed consents approved by the local institutional review board at Universidad de San Martín de Porres, Lima, Peru, and Federal Wide Assurance for the International Protection of Human Subject 0001532 were obtained. Individuals from Cajamarca, Peru, Ecuador and Cusco, including the districts of San Sebastian, San Jerónimo, Anta, Calca, Paucartambo and Pacarictampu (Fig. 1), data from the South American Genographic Database and those published elsewhere (Baca et al. 2012; Sandoval et al. 2013b, 2016; Roewer et al. 2013) were included in this study for comparison purposes.

### Analysis of Y-chromosome markers

The initial patrilineal analysis involved genotyping five Y-SNPs identified in South American natives, including M130, M242, M346, L54 and M3, using *Taq*Man assays (ABI) and a 7900HT Fast Real-Time PCR System (ABI), and 17 Y-STRs (Karafet et al. 2008; Jota et al. 2011). Subsequently, additional analysis of 64 Y- SNPs was used to refine several paternal lineages (Jota et al. 2016). PCRs for Y-STRs were performed as described (Sandoval et al. 2013b). The PCR products were subjected to capillary electrophoresis using ABI 3130XL Genetic Analyzer (Applied Biosystems), and STR alleles were genotyped using GeneMapper ID v3.2 software (Applied Biosystems, Foster City, California, USA). The DYS389b allele scoring was performed by subtracting DYS389I from DYS389II, and the DYS385 marker was not included in the statistical analyses.

### Analysis of mtDNA markers

Matrilineal analysis was performed using PCR amplification of the complete mtDNA control region (16,024–16,576 bp) corresponding to the revised Human Mitochondria Cambridge Reference Sequence (rCRS) (Andrews et al. 1999). Sequencing was performed as described (Sandoval et al. 2013b) using ABI 3130XL Genetic Analyzer (ABI) and Big Dye Terminator v.3.1. DNA sequences were aligned using SeqScape 2.6 (Applied Biosystems), and major haplogroups were assigned using MitoTool (Fan and Yao 2011) or haplogroup prediction tool from the Genographic Project (Behar et al. 2007). Indels and hotspot sites at nucleotide positions 303–315; 515–522; 16,182–16,193 and 16,519 were excluded from the statistical analyses.

### Statistical analyses

To analyse the phylogenetic relationship among individuals, median-joining algorithm of Network v.5.0.0.1 was used as described at the Fluxus Engineering website (http://www.fluxus-engineering.com) (Bandelt et al. 1999). Additionally, GenAlEx v.6.503 (Peakall and Smouse 2006) and mtDNA GeneSyn v.1.0 software (Pereira et al. 2009) were used for data conversions. In some cases, the Y-chromosome haplogroup assignment was corroborated using Bayesian approach with Hapest5 (Athey 2006) and the GenoChip 2.0 DNA Ancestry Kit (Elhaik et al. 2013) customised in Family Tree DNA (http://www.familytreedna.com). Principal components analysis (PCA) was used for clustering the 17 Y-STR haplotypes using the FactoMineR v. 1.00 package of R (http://www.r-project.org).

To calculate the time to the most recent common ancestor (TMRCA) in a group of STR haplotypes, the average squared difference and its inferences, as proposed by Ethio Helix calculator (https//ehelix.pythonanywhere.com/), were used.

## Results

### Y-chromosome results

#### Y-STR genealogy delineates a complex structure into two clusters of patrilineal inheritance among Panakas families

For comparison, we included approximately 1200 samples from the South American Genographic Database and from previously published studies; however, for clarity, we selected 282 individuals with 184 Y-STR haplotypes of the Q haplogroup phylogenetically related to the Panakas. Other haplotypes belonging to non-Q haplogroups (R, E, I and T) were not considered, as they represent Eurasian lineages and were probably identified due to post-Columbian admixture.

**Fig. 2:**
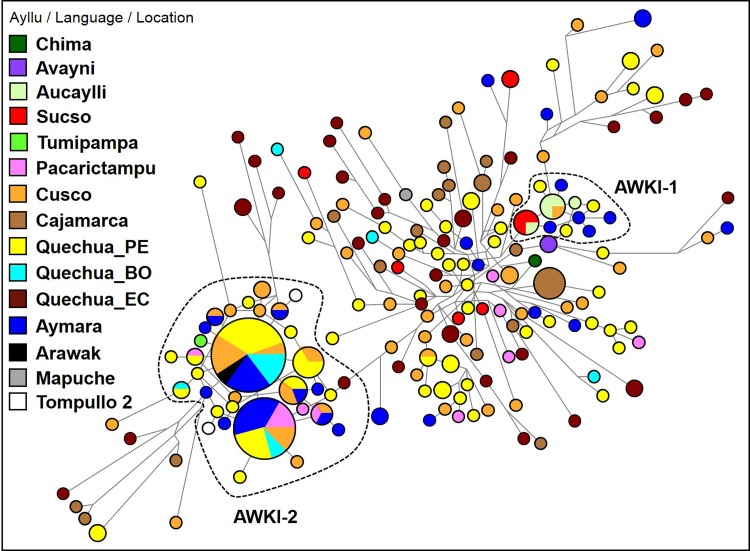
Median-joining network for Q-M3* Y-STR haplotypes among selected individuals (*n* = 282) from different populations. Different population groups (Ayllu/Language/Location) are indicated using distinct colours. Haplotypes composed of alleles on 15 Y-STRs are represented with circles; the size of the circle is proportional to the number of individuals, and the branch length is proportional to STR mutation steps (one-step unit between haplotypes in the AWKI-1 clan). The AWKI-1 and AWKI-2 clusters are identified as Q-M3 and Q-Z19483 lineages, respectively. Population groups: Chima, Avayni, Aucaylli, Sucso and Tumipampa are *ayllus* from San Sebastian and San Jerónimo; Peruvian Quechuas (Quechua_PE); Bolivian Quechuas (Quechua_BO); Ecuadorian Quechuas (Quechua_EC); and Argentinian Mapuche (Mapuche). Sample codes are listed in Supplemental Table 3a

The Y-STR haplotypes identified among the San Sebastian–San Jerónimo (SsSj; *n* = 28) and Pacarictampu (*n* = 12) populations are listed in Supplemental Table 3 (49 individuals in Supplemental Table 3a and 282 individuals in Supplemental Table 3b). The haplotype distribution of Y-STRs of the Panakas in the network indicated a high genetic differentiation among them (Fig. 2). However, two majoritarian clusters, AWKI-1 and AWKI-2, and several scattered Y chromosomes were observed, precluding the presence of a unique or more frequent patrilineal pattern.

Out of 18 genotyped individuals of 12 Panakas families, 8 individuals from 5 different families showed the closest Y-STR haplotypes to each other (AWKI-1 cluster). Among these eight individuals, individuals from the *ayllu* Sucso (K3, K11 and K12; *n* = 3) and an individual from *ayllu* Aucaylli (K22) shared a haplotype, which was related by a one-step mutation to individuals from the *ayllu* Aucaylli (K9, K37, K40; *n* = 3) that shared another Y-STR haplotype with an individual (Q_Cus03) from Maras District, Urubamba. Additionally, individual K36 was closely related by a one-step mutation to members of the corresponding *ayllu* Aucaylli (Fig. 2). On the contrary, two individuals (K35 and K41) from *ayllu* Avayni shared the same haplotypes and were related to Sucso–Aucaylli families, despite some step mutations. The other families were characterised by unrelated haplotypes and were not included in the AWKI-1 cluster. Among these unrelated haplotypes, two individuals from *ayllu* Sucso (K6 and K38) shared the same haplotypes.

At the opposite side of the phylogenetic tree was the AWKI-2 cluster, which included one individual (K34) from the *ayllu* Tumipampa of the Panaka family that was connected by a one-step mutation to the most frequent haplotype in the Andes (code = 140; Supplemental Table 3b). A total of 32 individuals from different Andean locations, including San Sebastian–San Jerónimo, Aymara- and Quechua-speaking individuals from the Altiplano region (Peru–Bolivia) and other localities of the Cusco region, Arequipa, Junín, Apurimac and Amazonian Machiguengas shared this haplotype. Additionally, this common haplotype was connected by a one-step mutation with another haplotype (code = 141, Supplemental Table 3b) that was shared by 24 individuals from the same regions mentioned above, except Apurimac. Furthermore, two haplotypes (T2CH13 and T2CH81) of ancient DNA samples from the Tompullo 2 site (Inka, 1500 AD) of the Arequipa region (Baca et al. 2012) were connected by a two-step mutation to the cluster AWKI-2 (T2CH13 to K2, T2CH81 to K18). A similar genetic profile for the Y-STRs among all individuals from San Sebastian, San Jerónimo and Pacarictampu (*n* = 49) was obtained using PCA (Supplemental Fig. 1).

#### Genetic link to Southern Andes of Peru

We observed that the AWKI-1 cluster also included five Aymara-speaking individuals from the Anapia Island and the Santa Rosa de Yanaque community of Lake Titicaca and three Quechua-speaking individuals from the Colca Canyon and Chuquibamba in Arequipa and Ayacucho, respectively. Thus, the AWKI-1 cluster mainly comprised individuals from the Lake Titicaca region and other southern locations, including Arequipa, located southwest of Cusco. On the contrary, the AWKI-2 cluster, which included K34, comprised seven individuals from Pacarictampu (K2, K24, K26, K27, K30, K31 and K49) and one from Puno.

#### Coalescence time among haplotypes of AWKI-1 and AWKI-2 clusters

The evolutionary models of mean TMRCA over multiple generations using the Zhivotovsly mutation rates (Z-TMRCA) and using all available pedigree mutation rates (P-TMRCA) were calculated, and the most probable TMRCA for three closely related haplotypes (individuals K3, K9 and K36 in AWKI-1 cluster, and individuals K34, K19 and K24 in AWKI-2 cluster) was obtained. According to the P-TMRCA model, the common paternal ancestor for Sucso–Aucaylli families (included in the AWKI-1 cluster) was predicted to have occurred approximately 18 generations or 540 years ago (considering 30 years per generation). In the AWKI-2 cluster, which included one individual from ayllu Tumipampa (K34) and two individuals from San Sebastian (K19) and Pacarictampu (K24), the TMRCA was estimated to occur approximately 30 generations or 900 years ago (Supplemental Table 3c).

### mtDNA results

#### Heterogeneity of maternal lineages among the Panakas

We identified four most common Native American mtDNA lineages as A2, B2, C1 and D1 and a rare maternal lineage D4h3 among others like M17a, a common lineage observed in Southeast Asia. The distribution of mtDNA lineages among the Panakas families and other individuals from San Sebastian–San Jerónimo and Pacarictampu (*n* = 51) showed a higher frequency of B2 (*n* = 29) such as that observed in many Andean populations (Sandoval et al. 2013b). Among other lineages, the distributions were C1 (*n* = 9), D1 (*n* = 6), A2 (*n* = 5), D4h3a (*n* = 1) and M17a (*n* = 1); their mtDNA SNPs relative to rCRS are listed in Supplemental Table 4.

**Fig. 3:**
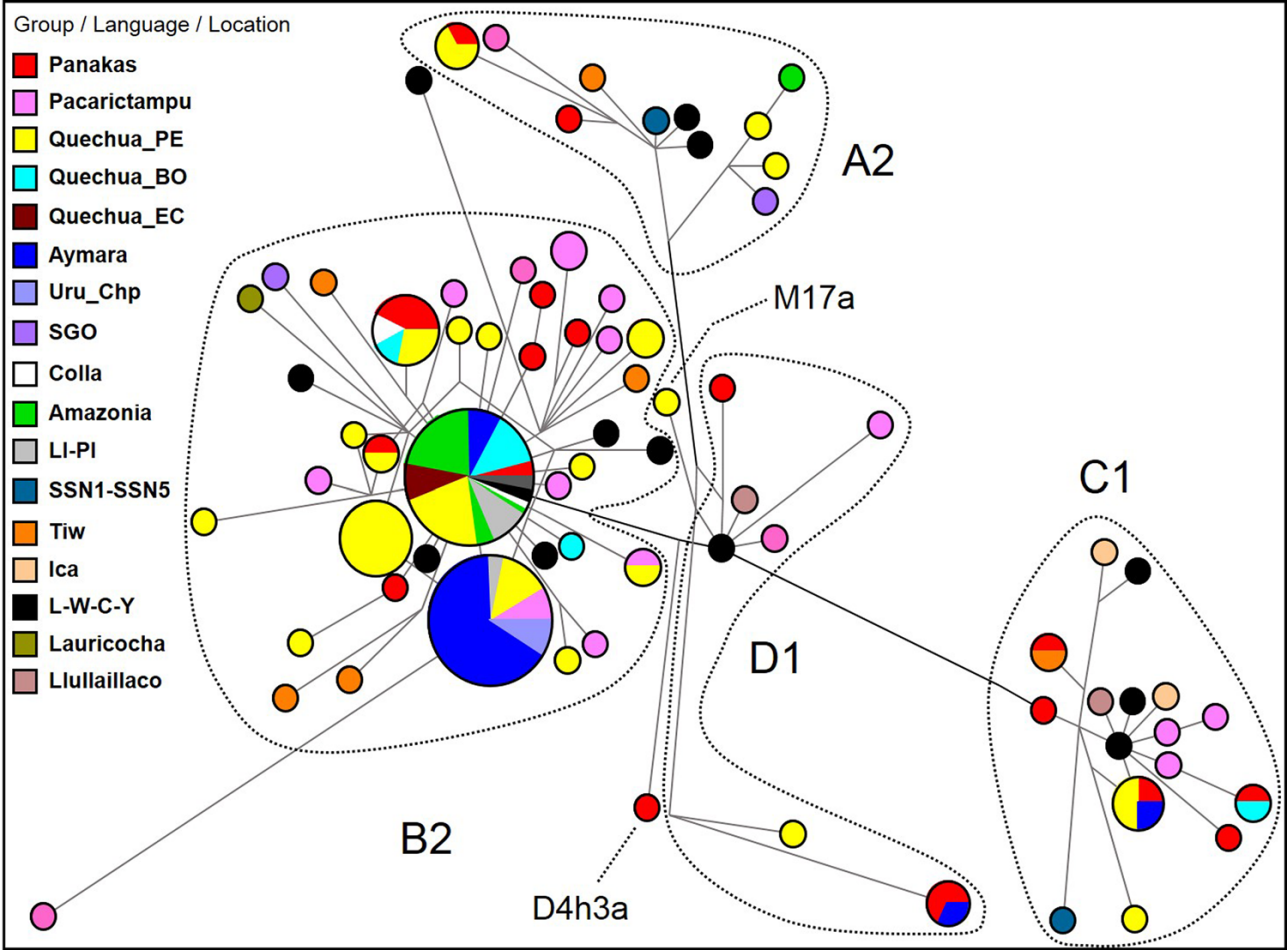
Median-joining network for A2, B2, C1 and D1 control region mtDNA haplotypes among selected individuals (*n* = 193) from different populations. Different population groups (Group/Language/Location) are indicated using distinct colours. The mtDNA haplotypes are indicated with circles; the size of the circle is proportional to the number of individuals, and the branch length is proportional to the number of nucleotide changes. Population groups: the Panakas comprised individuals from all five *ayllus* of the San Sebastian and San Jerónimo districts; Peruvian Quechuas (Quechua_PE); Bolivian Quechuas (Quechua_BO); Ecuadorian Quechuas (Quechua_EC); Urus from Chipaya, Bolivia (Uru_Chp); Lima and Piura, Peru (LI-PI); SGO samples were from Santiago del Estero, Argentina (Pauro et al. 2013); Colla samples were from Jujuy, Argentina (Álvarez-Iglesias et al. 2007); SSN1–SSN5, Tiw (Tiwanaku), Ica, Llullaillaco and L–W–C–Y (Lima–Wari–Chancay–Ychsma) samples were from Llamas et al. (2016); and Lauricocha sample was from Huanuco (Fehren-Schmitz et al. 2015). Amazonia comprised samples from several Amazonian ethnic groups, including Andoas and Jivaro (Peru), Arawak, Tupi-Guarani, Cayubaba, Itonama and Movima (Bolivia) and Je and Puinavean (Brazil)

First, the haplotypes of autochthonous mtDNA lineages (A2, B2, C1 and D1) of the Panakas were compared with South American Genographic Database (with 2335 selected individuals from different populations), including published data (Álvarez-Iglesias et al. 2007; Pauro et al. 2013; Fehren-Schmitz et al. 2015; Valverde et al. 2016; Llamas et al. 2016). To simplify the phylogenetic reconstruction, a group of closest haplotypes (*n* = 193) was selected (Fig. 3**)**. In general, our analysis showed a close genetic relationship of the Panakas with native populations located south of Cusco in Peru and Bolivia.

Among the B2 haplotypes, we observed two major groups of the closest haplotypes (73-263-499-16217 and 73-186-263-499-16217), which were shared by 70 individuals (including 4 from ancient DNA samples) and 23 individuals, respectively. The first group included individuals from different regions/languages of Peru, Bolivia, Ecuador and Brazil (Fig. 3). Additionally, three individuals from *ayllus* Sucso (K6 and K7) and Avayni (K41) were included in this group. The ancient DNA samples were from the pre-Inka cultures of Lima and Chancay (ACAD10789 and ACAD11200 samples, respectively; Llamas et al. 2016) and Ychsma (ACAD10713 and ACAD10720 samples; Valverde et al. 2016). The second group of shared haplotypes included mostly Aymara- and Quechua-speaking individuals from the Altiplano region as well as K25 and K26 from the Pacarictampu District. On the contrary, seven individuals shared the haplotype 73-204-207-263-499-16217, including three from *ayllus* Chima (K13 sample), Sucso (K33 sample) and Aucaylli (K36 sample), three Quechua-speaking individuals from Peru and Bolivia and a Colla individual from Jujuy/Salta provinces of Argentina (CO-07 sample; Álvarez-Iglesias et al. 2007).

In the A2 lineage, we observed a shared haplotype between K12 (from *ayllu* Sucso) and two individuals from the Colca Canyon (Arequipa). The C1 lineage included two shared haplotypes, one shared between K15 from Cusco, two individuals from Apurimac and one Aymara-speaking individual from Puno and another shared between K22 (from *ayllu* Aucaylli) and a Quechua-speaking individual from Bolivia. Surprisingly, a shared haplotype between K39 (from *ayllu* Sucso) and an ancient sample (ACAD13241) from Tiwanaku period dated 962 years ago (Llamas et al. 2016) was also identified. The D1 lineage included a shared haplotype between two samples from Cusco, K4 (from *ayllu* Sucso), K19 and an Aymara-speaking individual from Bolivia. On the contrary, an individual from Panakas (labelled as K11 sample from *ayllu* Sucso) belonged to the D4h3a lineage, which is found in different regions of South America (Catelli et al. 2011; Sevini et al. 2013; Gómez-Carballa et al. 2016) and shared a haplotype with a Quechua-speaking individual from Apurimac (sample Tor676, from Perego et al. 2009) (Supplemental Fig. 2).

## Discussion

Most chroniclers and historians state that the Inka lineage, even before the migration to the Cusco valley, inherited power in a patrilineal manner (Cobo 1653; Espinoza-Soriano 1997). Although no unique patrilineal Inka descent was determined from the genetic analysis of 18 individuals belonging to putative Panakas families, two peculiar Y-STR clusters were identified.

The AWKI-1 cluster (a Q-M3* lineage) comprised a group of eight individuals from five Panakas families with a documented genealogical and historical link to the *ayllus* Sucso and Aucaylli from San Sebastian and San Jerónimo localities of Cusco. Our data suggest that a common ancestor of closely related individuals (K3, K9 and K36) lived approximately 18 generations ago within the period of the Inka Empire (around 1400 AD). In addition, several Aymara-speaking individuals were included in the AWKI-1 cluster. We also observed a close parentage between individuals from *ayllu* Sucso–Aucaylli (Cusco) and from the Lake Titicaca region, which was consistent with chronicles and linguistic sources (de la Vega 1609; Domínguez-Faura 2010; Cerrón-Palomino 2013). The closest observed haplotypes among two putative imperial *ayllus* from Cusco and individuals from Anapia (an island close to *Isla del Sol*, located in Lake Titicaca) might reflect a gene flow that occurred during the Inka expansion. Both the Inka Emperor Tupac Yupanqui and Paullu Inka had relatives in Copacabana and other towns around the Lake Titicaca Basin (de León 1553; Espinoza-Soriano 1972; Julien 2002).

On the contrary, AWKI-2, the second cluster of Y-STR haplotypes, belonged to a Q-Z19483, a sub-lineage of Q-M3, which probably expanded in the Late Intermediate Period: Wari-Tiwanaku or Inka (Jota et al. 2016). The calculated TMRCA of closely related haplotypes of the Q-Z19483 lineage (samples K34, K19 and K24) suggests that they share a common ancestor who probably lived about 30 generations ago (around 1000 AD), which is consistent with our previously reported mean TMRCA (Jota et al. 2016). We identified shared haplotypes of the AWKI-2 cluster comprising individuals from different populations of the Andes from Peru and Bolivia, which is in agreement with our previous study (Sandoval et al. 2013b) (Supplemental Fig. 3). Although patrilineal connection of the AWKI-2 cluster with Inka rulers cannot be directly supported, the association of the AWKI-2 cluster with a recently expanding Y sub-lineage in the last millennium is a remarkable finding.

Our results showed that the search for a unique patrilineal Y chromosome was a complex task, but possible by relying on the identified Panakas members. Clan identity (a social aspect) is independent of a direct patrilineal descent (genetic genealogy), as it is for any group in society. Large differences observed among haplotypes of the Panakas suggest that intervention in male lines occurred from at least the sixteenth century until the twentieth century and perhaps before this time. The intervention could also have resulted from extra-paternity, where the “official” father is not the same as the biological one, whose incidence is estimated from 1 to 30% per generation in the worldwide population (Lucassen and Parker 2001).

In this study, 18 individuals from the Panakas were tested; however, a larger sample size is desirable to corroborate the genetic results of the putative Inka rulers´ descendants. Another possible way to investigate the Y chromosome ancestry of the royal Inkas is by using DNA from the bodily remains of a recorded male-to-male descendant. For instance, remains of sons and grandsons of Huayna Capac (the last pre-colonial ruler) have been found buried in colonial churches, e.g. his son Paullu Inka, who was buried under the main altar of the church of San Cristobal in Cusco (Martín-Rubio 2009).

The maternal genealogical analyses of the Panakas indicated that they were descendants of different mitochondrial haplogroups (A, B, C and D). Moreover, a slightly homogeneous distribution of mtDNA A2, B2, C1 and D1 lineages was identified in the Andes for Quechua and Aymara speakers (Sandoval et al. 2013b), suggesting a high maternal gene flow among native Andean populations of Ecuador, Colombia, Peru, Bolivia, Chile and Argentina following the rituals of marriage and migration along the Andes in the Late Intermediate Period (Tarazona-Santos et al. 2001) and in colonial and republican periods.

With respect to the ancestral homeland of the royal Inkas, the genetic study of the Panakas families with putative Inka ancestry showed closer genetic affinity with Quechua- and Aymara-speaking populations from southern Peru and northern Bolivia, including areas of the previous Tiwanaku Empire, such as Lake Titicaca and the Altiplano. This was consistent with the hypothesis that the Inkas had ancestors from the Altiplano region and Pacarictampu (Cusco). However, well-recorded ancient DNA samples from the Inka and Tiwanaku cultures should be used to determine the direction and timing of the origin and dispersal of the imperial Inkas.

## Electronic supplementary material

Below is the link to the electronic supplementary material.

Supplementary material 1 (DOCX 1910 KB)

Supplementary material 2 (XLSX 9 KB)

Supplementary material 3 (XLSX 13 KB)

Supplementary material 4 (XLSX 16 KB)

Supplementary material 5 (XLSX 43 KB)

Supplementary material 6 (XLSX 11 KB)

Supplementary material 7 (XLSX 8 KB)

Supplementary material 8 (XLSX 13 KB)

Supplementary material 9 (XLSX 8 KB)
