# Supplementary material for: Genetic ancestry of families of putative Inka descent
Source: Mol Genet Genomics. 2018 Mar 3;293(4):873–81. doi: 10.1007/s00438-018-1427-4 (PMC6061041; doi:10.1007/s00438-018-1427-4)
Supplement: Supplementary file 1 — Supplementary material 1 (DOCX 1910 KB) [file 438_2018_1427_MOESM1_ESM.docx]

**SUPPLEMENTAL FIGURES**

**Genetic Ancestry of Families of Putative Inka Descent**

José R. Sandoval^1,2,*^, Daniela R. Lacerda^2^, Marilza S. Jota^2^, Ronald Elward^3^, Oscar Acosta^1^, Donaldo Pinedo^1^, Pierina Danos^1^, Cinthia Cuellar^4^, Susana Revollo^4^, Fabricio R. Santos^2^ and Ricardo Fujita^1^

^1^Centro de Investigación de Genética y Biología Molecular (CIGBM), Instituto de Investigación, Facultad de Medicina Humana, Universidad de San Martín de Porres, Lima, Perú.

^2^Laboratório de Biodiversidade e Evolução Molecular (LBEM), Instituto de Ciências Biológicas, Universidade Federal de Minas Gerais, Belo Horizonte, Brazil.

^3^Limaq Publishing SAC, Lima, Perú.

^4^Universidad Mayor de San Andrés (UMSA), La Paz, Bolivia.

* Corresponding author

**
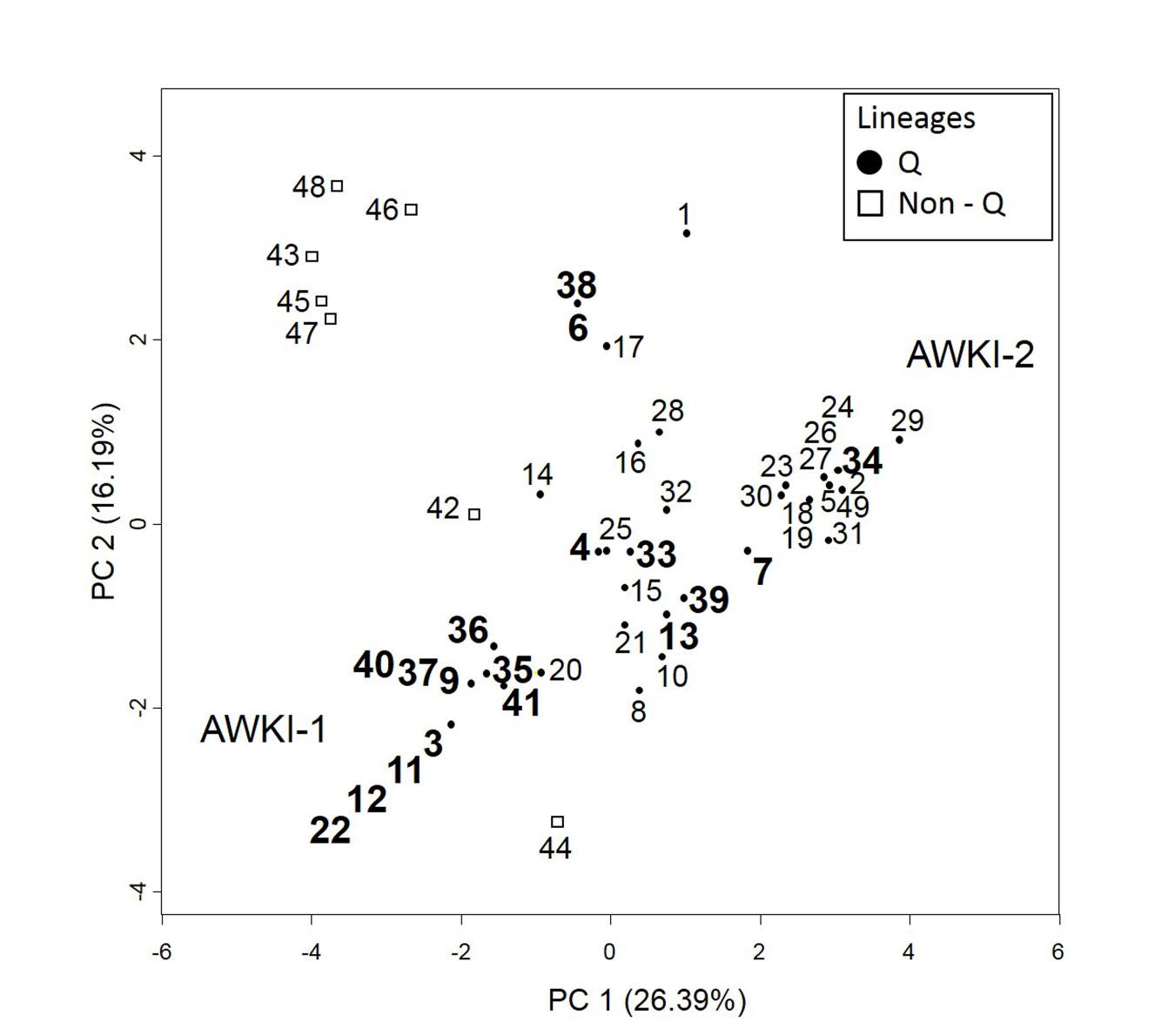
**

**Supplemental Fig. 1** Scatter plot of Principal Components Analysis (PCA) for the 17 STR of Y-chromosome among 49 individuals from the districts of San Sebastian-San Jerónimo and Pacarictampu in Cusco (Peru). The big numbers highlighted in bold are individuals who belong to Panakas families. See codes of individuals (adding prefix K to numbers) or their haplotypes in the Supplemental **Table 3a**.


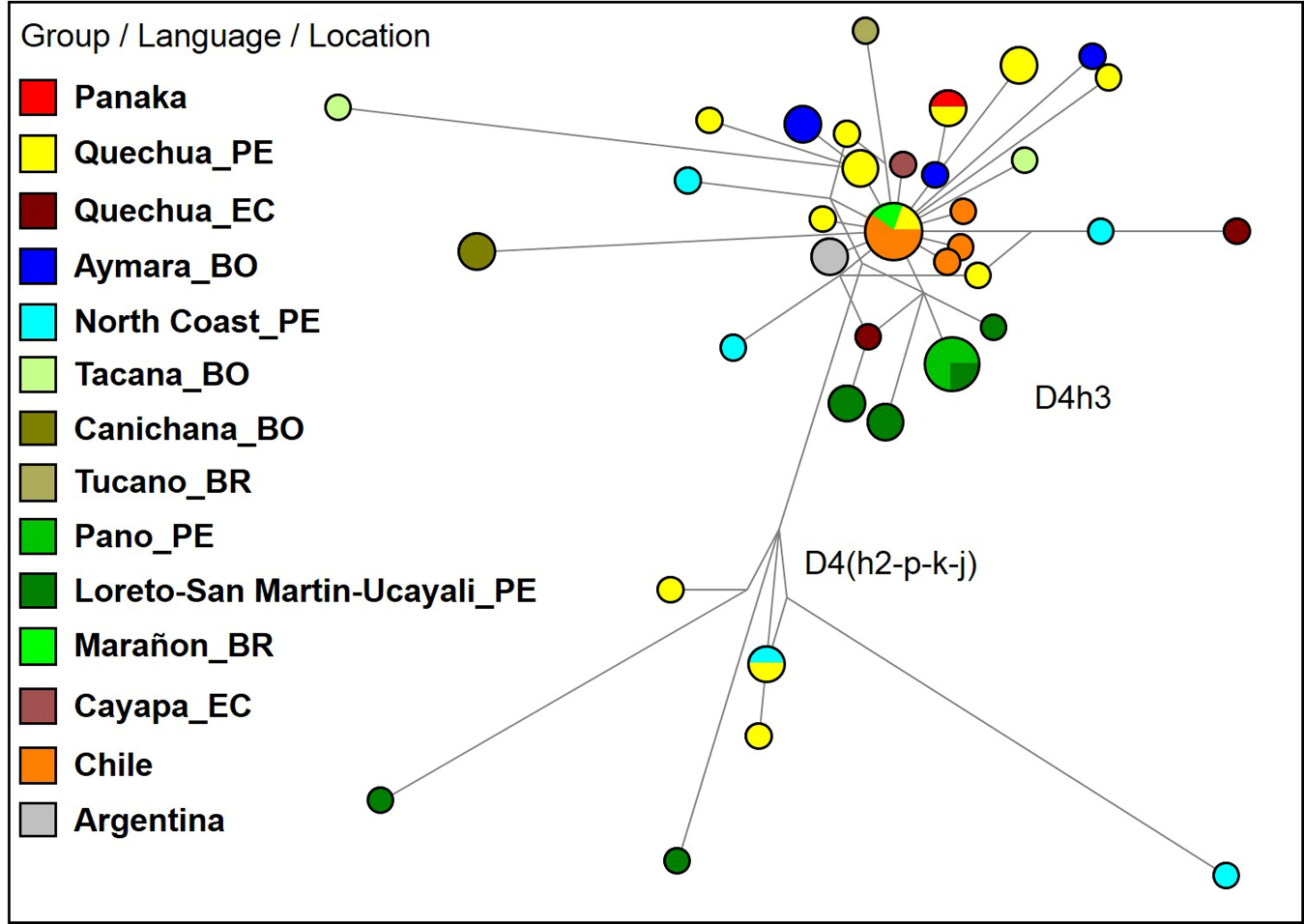


**Supplemental Fig. 2** Median-Joining network for the D4 lineage based on mtDNA control region sequences. In red: an individual from *ayllu* Sucso (K11). PE=Peru, EC=Ecuador, BO=Bolivia, BR=Brazil.


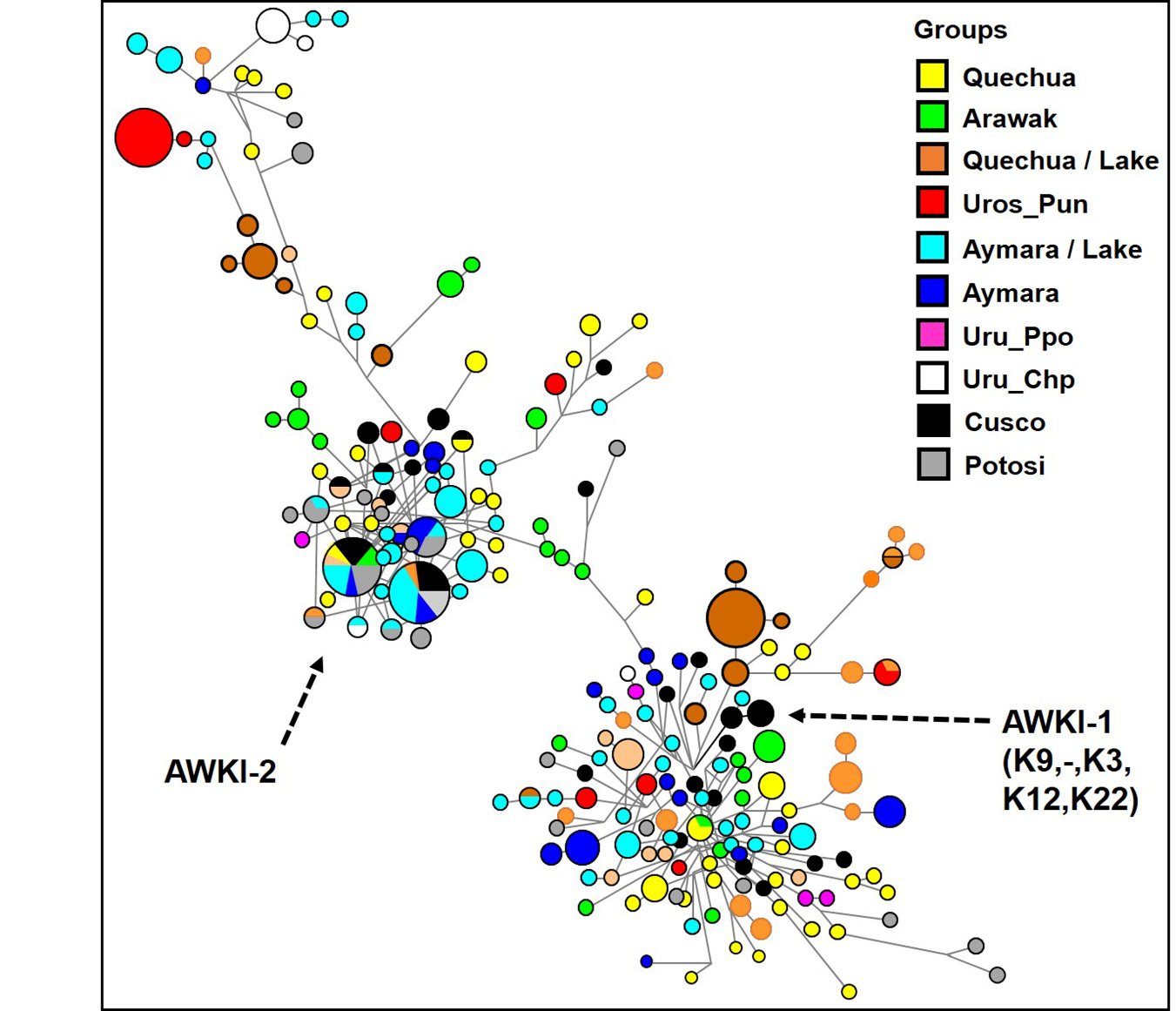


**Supplemental Fig. 3** Median Joining network for Q-M3 STR of Y-chromosome among 22 Peruvian and Bolivian populations (modified from the Fig. 2, Sandoval et al. 2013b). The haplotypes composed of alleles on 15 Y-STRs are represented by circles with a size proportional to the number of individuals, and branch lengths are proportional to STR mutation steps (one-step unit between haplotypes of AWKI-1 clan). Quechua / Lake includes individuals from Taquile (dark orange), Amantani (orange) and Capachica (light orange).
